# Single‐Cell Transcriptome‐Wide Mendelian Randomization and Colocalization Uncover Potential Immunocytes‐Related Therapeutic Targets for Obesity

**DOI:** 10.1111/jcmm.71185

**Published:** 2026-05-12

**Authors:** Xingjian Zhang, Yong Tian, Xiaoying Ren, Juan Tian, Jia Liu, Guang Wang

**Affiliations:** ^1^ Department of Endocrinology, Beijing Chao‐Yang Hospital Capital Medical University Beijing People's Republic of China

**Keywords:** drug target prioritization, immune cells, Mendelian randomization, obesity, single‐cell eQTL

## Abstract

Weight‐loss treatment is crucial for individuals with obesity to prevent various complications. The role of Immune cells in obesity has been recently recognized, whereas its translation into therapy requires identifying key target genes. We performed Mendelian randomization (MR) analysis to assess causal relationships between expression quantitative trait loci (eQTL) of 14 immune cells and obesity‐related traits (obesity, body mass index and body fat percentage), and validated the results in colocalization analysis. For the putative causal genes identified by the MR and colocalization analyses, we conducted pathway enrichment, differential expressed gene (DEG) analysis and search of druggable evidence, and utilized a Tier system to prioritize drug targets for obesity. MR and colocalization evidence was observed for 1630 genes associated with one or more obesity‐related traits, mainly expressed in CD4^+^ naive/central memory T cells and enriched in antigen processing and presentation pathways. Forty‐one genes showed causal relationship with all three outcomes, among which 19 genes have not been reported for obesity previously. DEG analysis using single‐cell RNA sequencing data of blood or adipose tissue indicated that the differential expression of *UBE2Z* in monocytes, *ZCCHC7* in T cells, and *FNBP4* in B cells between lean and obese individuals were consistent with the MR results. By searching drug‐gene interaction databases, we found targeted drugs for *PYGB* and *PRUNE1*, and *PYGB* was the top gene ranked in the Tier system. This study provides evidence for the involvement of immune cells in obesity, and the potential cell‐specific, immune‐related targets for obesity treatment.

## 1 Introduction

Obesity, the excessive accumulation of adipose tissue (AT), has been a significant public health challenge worldwide. Obesity is associated with adverse health outcomes throughout the life course such as reduced life expectancy and comorbidities such as cardiovascular disease and type 2 diabetes (T2D) [1]. A loss of 5%–10% of body weight reduces lipids, blood pressure and glycated haemoglobin, and improves insulin sensitivity [2], indicating a need for effective weight‐loss treatment.

The tools of obesity management include lifestyle interventions, anti‐obesity medications and bariatric surgery. Most available anti‐obesity therapies act on central appetite pathways, and advances have also been made in the development of medications targeting monogenic obesity [3]. However, pharmacotherapy may be limited by tolerability, accessibility, or weight regain after discontinuation, and surgery, although effective, is not suitable or acceptable for all patients. In recent years, chronic low‐grade inflammation and immune dysregulation have been recognized as being associated with metabolic disorders such as obesity and T2D [4], and immune pathways have emerged as potential targets for intervention. Previous studies have highlighted the role of immune cells in the links between sympathetic neuron signals and the orchestration of metabolism and obesity in animal models [5]. Furthermore, type 1 innate lymphoid cells were found to be involved in the mechanisms by which gut microbiota mitigate obesity [6]. However, most evidence is preclinical and mechanistic, whereas studies evaluating immunomodulatory interventions for obesity treatment remain limited. This gap may be partly attributable to the complex and heterogeneous immune landscape of obesity, tissue‐specific immune functions, and concerns about target selectivity and systemic safety.

The translation of the aforementioned findings into actionable drug targets requires the identification of both causal genes and the specific cell types in which the genes exhibit their functions. Genome‐wide association studies (GWASs) have been widely used to find out genomic loci associated with human disease [7]. Using genetic variants from GWAS data as instrumental variables (IVs), Mendelian randomization (MR) has shown its advantages in inferring putative causal effects of the expression levels of genes and proteins on outcomes in a large number of studies, making it helpful in prioritizing drug targets for various diseases [8, 9]. However, most GWAS studies examine gene expression regulated at the bulk tissue level. Yazar et al. [10] generated the single‐cell RNA sequencing (scRNA‐seq) data from 1.27 million peripheral blood mononuclear cells (PMBCs) collected from 982 donors and identified the cell‐type specific expression quantitative trait loci (eQTLs) in each of 14 immune cell types, providing an unprecedented opportunity to understand the complex interplay between the gene expression in different types of immune cells and diseases. Taking advantage of single‐cell eQTLs (sc‐eQTLs), sc‐eQTL MR helps reduce cellular heterogeneity and enhance mechanistic resolution, elucidating the underlying causal effects that are usually masked by the conventional bulk eQTL MR. The sc‐eQTL MR has been applied in studies on COVID‐19, atopic dermatitis, cardiometabolic diseases, and Parkinson's disease, providing a new approach for detecting potential mechanisms and therapeutic targets for diseases at the cell‐type level [11, 12, 13, 14]. Notably, the study by Hong et al. [13] has applied findings from sc‐eQTL MR to drug target prioritization using molecular docking. Taken together, sc‐eQTL MR could be a new method that helps investigate immune‐related mechanisms and potential target genes for obesity at a cell‐specific level.

**FIGURE 1:**
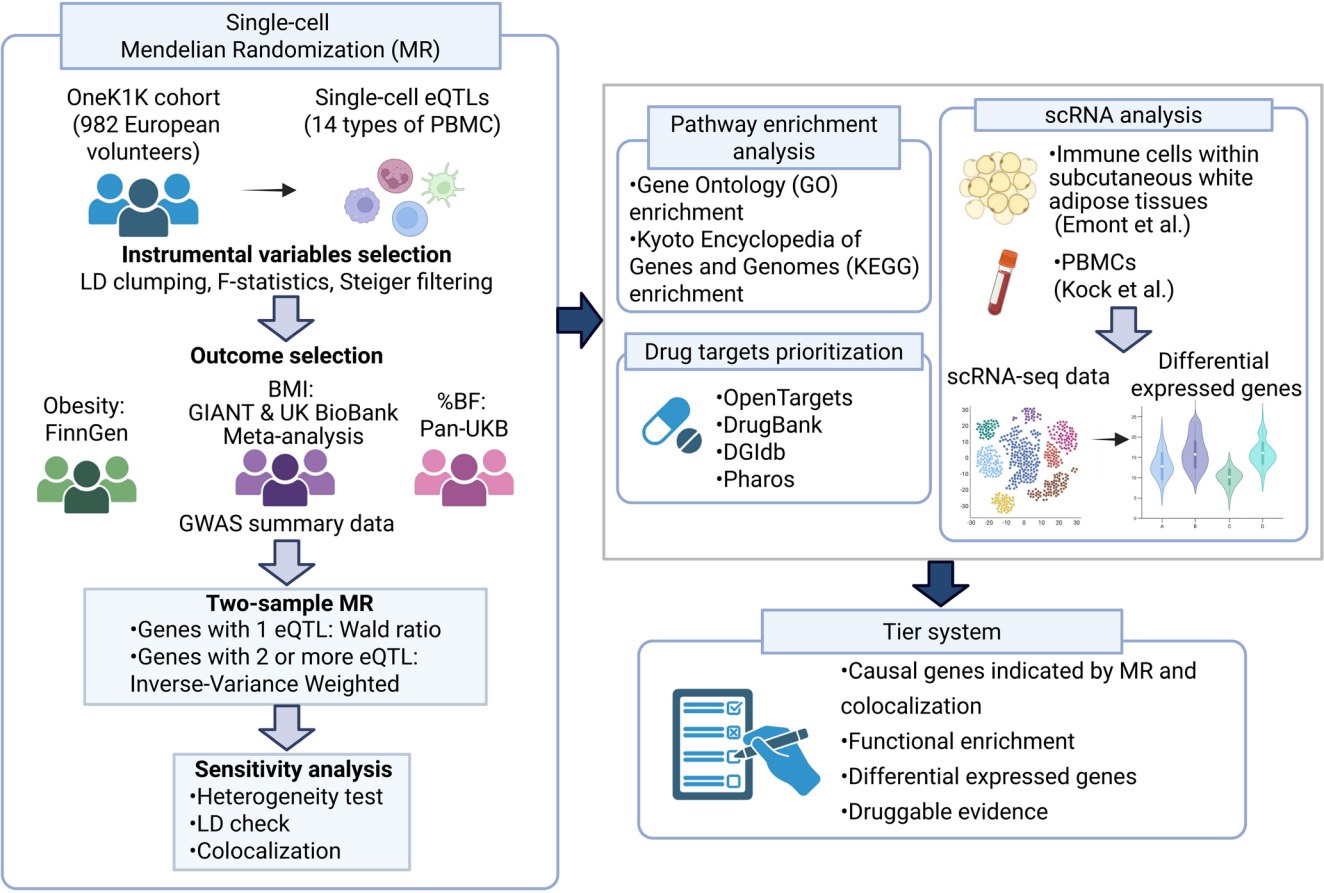
Overview of the study workflow (created by BioRender).

In this study, we conducted single‐cell transcriptome‐wide MR and colocalization analyses to investigate the causal associations between the cell‐specific gene expressions in 14 immune cell types and the obesity‐related traits including obesity, body mass index (BMI) and body fat percentage (%BF). For the causal genes identified by MR and colocalization, we further performed functional enrichment analysis, as well as differentially expressed genes (DEG) analysis using the scRNA‐seq data of blood and adipose tissues from other studies. After searching for drugs related to the causal genes, we used a Tier system integrating genetic evidence, bioinformatic findings, and drug repurposing to prioritize potential drug targets for obesity. The study design is shown in Figure 1.

## 2 Materials and Methods

### 2.1 Genetic Instrumental Variables Selection

In this study, we selected instrumental variables from the summary‐level sc‐eQTL data of peripheral blood mononuclear cells (PBMCs), which were obtained from the OneK1K database (https://onek1k.org/). We selected the eQTLs associated with gene expression at a *p* value < 0.005 based on previous research [12]. The eQTLs in linkage disequilibrium (LD) with the top association signals (LD *r* ^2^ < 0.01) were removed in the LD clumping. To ensure sufficient instrument strength of the eQTLs, we calculated the *F*‐statistics of each instrument. Furthermore, we performed Steiger filtering to test the directionality of the eQTL‐outcome associations [15]. Only eQTLs with *F*‐statistics > 10 and consistent directionality were retained as instruments.

### 2.2 Outcome Selection

We selected GWAS summary statistics of obesity from the FinnGen database (https://www.finngen.fi/) R12 release, which comprises 31,499 cases and 468,693 controls of European ancestry, published by Kurki et al. [16]. The GWAS meta‐analysis summary statistics of BMI (*n* = 484,680) were extracted from the Genetic Investigation of Anthropometric Traits (GIANT) and UK BioBank meta‐analysis [17], and the GWAS data on %BF was collected from the Pan‐UK Biobank (*n* = 432,262) [18].

### 2.3 Single‐Cell eQTL MR Analysis

In the MR analysis, we aimed to assess the potential causal effects of gene expressions at the single‐cell level on obesity using the cell‐specific eQTLs and the GWAS data of obesity‐related traits. The methods of MR analysis applied in our study were dependent on the number of eQTLs used as instrumental variables for each gene. We utilized the Wald ratio method for the genes with only one eQTL, and the Inverse Variance Weighted (IVW) method for the genes with two or more eQTLs [12, 19]. To account for multiple testing, *p* values from MR analyses were adjusted using the Benjamini–Hochberg false discovery rate (FDR) procedure, which was applied within each cell type–trait combination. The MR estimates with a FDR–adjusted *p* value less than 0.05 were considered to show robust signals and selected for follow‐up analyses. To further enhance robustness of the findings, these candidate signals were subsequently evaluated using the following colocalization analysis and consistency across multiple obesity‐related traits, and only those supported by multiple lines of evidence were prioritized as high‐confidence candidate genes. The MR analysis was conducted using R version 4.3.3 along with the R package TwoSampleMR (version 0.6.8) [20].

### 2.4 Colocalization Analysis

To increase the credibility of the MR results, we applied sensitivity analyses of the gene–outcome MR signals. First, we performed Cochran's *Q* test for IVW results to detect the potential heterogeneity of MR estimates, and the gene‐outcome signals with evidence of heterogeneity (*Q* value < 0.05) were excluded from the candidates for further analyses.

For the MR signals that passed the heterogeneity test, we conducted LD check and genetic colocalization analysis to obtain more reliable colocalization evidence [21]. The LD check method was used to estimate the LD *r* ^2^ between each eQTL and GWAS signals in the region associated with the outcomes, where *r* ^2^ ≥ 0.7 was considered evidence of approximate colocalization [14]. Then we employed the ‘coloc’ R package (version 5.2.3) for colocalization analysis to detect the shared causal variants influencing obesity‐related outcomes within 100 kb upstream and downstream of each candidate eQTL. Colocalization analysis provides the posterior probabilities (PPs) of five hypotheses: absence of associations with either of the traits (H0); associations exclusively with one of the traits (H1); associations exclusively with the other trait (H2); associations with both traits but driven by different causal variants (H3); and associations with both traits, driven by the same causal variant (H4) [22]. Significant colocalization was defined as (PP.H3 + PP.H4) > 0.8 and [PP.H4/(PP.H3 + PP.H4)] > 0.9, indicating that the two genetic association signals (for gene expression and outcome) are likely to share the same causal variant [23]. The prior probabilities were set to *p*_1_ = 1 × 10^−4^, *p*_2_ = 1 × 10^−4^ and *p*_12_ = 1 × 10^−5^. If either the LD check or the colocalization analysis passed the test, it was considered evidence supporting the colocalization relationship [12].

### 2.5 Sensitivity Analyses

Considering the relatively relaxed threshold for IV selection and colocalization analysis, we conducted a series of sensitivity analyses to improve the robustness of our results. For the candidate genes derived from the above analyses, we repeated MR analyses with a stricter threshold for IVs (*p* < 5 × 10^−6^). Then we set a more rigorous standard (PP.H4 > 0.8) to further identify genes with significant evidence of colocalization. Evidence from these supplementary analyses was integrated into the Tier system for potential target genes described below.

### 2.6 Pathway Enrichment Analysis

To identify the functional pathways that were enriched by the candidate causal genes, we performed Gene Ontology (GO) and Kyoto Encyclopedia of Genes and Genomes (KEGG) enrichment analyses (*p* value cutoff = 0.05) using the clusterProfiler R package (version 4.10). Visualization of the pathway enrichment results was conducted using the ggplot2 R package (version 3.5).

### 2.7 scRNA‐Seq Analysis in Human PBMCs and Adipose Tissue

We performed scRNA‐seq analyses supportive of MR and colocalization. The PBMC scRNA‐seq data included PBMC samples of 20 donors from the study by Kock et al. [24], which was downloaded from CELLxGENE (https://cellxgene.cziscience.com). We also obtained the publicly available scRNA‐seq data of immune cells from subcutaneous white adipose tissue (SAT) of 8 donors, which was reported by Emont et al. [25]. For the PBMC data, 10 samples from non‐obese donors (BMI < 30) and 10 samples from obese donors (BMI ≥ 30) were selected for analysis (Table S1), whereas for the data of SAT, we selected scRNA‐seq data from 4 non‐obese donors (BMI < 30) and 4 obese donors (BMI > 40) for analysis (Table S2).

For each dataset, we used the Seurat R package to process the data and filtered out cells with detected genes outside the range of 200–8000 and cells with mitochondrial gene content exceeding 25% of total gene expression. We subsequently performed data normalization, followed by dimensionality reduction and clustering based on the principal component analysis (PCA) and the uniform manifold approximation and projection (UMAP). Cell types were then annotated based on the expression of marker genes of PBMCs and SAT immune cells, respectively [25, 26, 27]. Using the FindMarkers function of the Seurat package for detecting differential gene expression and Bonferroni multiple testing correction method for *p* value adjustment, we identified DEGs (adjusted *p* value < 0.05) between the lean and obese groups.

### 2.8 Drug Targets Prioritization

We selected the genes showing evidence of causal relationships with all three obesity‐related traits for the assessment of potential drug targets. We searched the genes on OpenTargets (https://platform.opentargets.org), DrugBank (https://go.drugbank.com), Drug–Gene Interaction database (https://www.dgidb.org), Pharos (https://pharos.nih.gov) [28] and literature of related drug trial studies, to identify genes with druggability or opportunities for drug repurposing. Finally, to evaluate the potential drug targets for obesity treatment, we developed a Tier system based on previous studies [12] and the findings of our study. This Tier system integrates evidence from functional enrichment analysis, differentially expressed genes (DEGs) identified from scRNA‐seq data, and druggability assessments. Details of the scoring scale are provided in Table 1. To assess the robustness of the prioritization framework, we performed sensitivity analyses using alternative scoring schemes, including an equal‐weight model across evidence domains, and a model with reduced weights for annotation‐dependent components (functional enrichment, scRNA‐seq evidence and druggability). Ranking stability was evaluated by the persistence of top‐ranked genes and Spearman rank correlation between the original and alternative rankings.

**TABLE 1:** Systematic prioritization for putative novel causal genes using four lines of evidence.

Cell type	Gene	MR	Colocalization	Score of functional enrichment	Score of scRNA‐seq evidence	Druggable evidence	Total score	Causal gene prioritization
		Number of exposure‐outcomes (*X*) significant in sensitivity analysis of MR^a^: *X* = 1, score = 0.1; *X* = 2, score = 0.3; *X* = 3, score = 0.5	Number of exposure‐outcomes (*X*) significant in sensitivity analysis of colocalization^b^: *X* = 1, score = 0.1; *X* = 2, score = 0.3; *X* = 3, score = 0.5	Number of enrichment pathways (*X*): *X* < 5, score = 0.1; 5 ≤ *X* < 15, score = 0.3; *X* ≥ 15, score = 0.5	Evidence of DEG in blood or adipose, score = 0.3	Genes with available drugs, score = 1; only druggable genes, score = 0.5; lacking druggable evidence, score = 0		Tier 1: score ≥ 1; Tier 2: 0.5 ≤ score < 1; Tier 3: score < 0.5
CD4 NC	PYGB	0.5	0.5	0.3	0	1	2.3	Tier 1
CD4 NC	ZCCHC7	0.5	0.5	0.3	0.3	0	1.6	Tier 1
CD8 ET, CD8 NC	DDX42	0.5	0.3	0.3	0	0.5	1.6	Tier 1
CD8 ET	PRUNE1	0	0.1	0.3	0	1	1.4	Tier 1
CD4 ET	FTSJ3	0	0.3	0.5	0	0.5	1.3	Tier 1
B Mem, NK R	FNBP4	0.5	0.5	0	0.3	0	1.3	Tier 1
CD8 ET	SPN	0	0.5	0.5	0	0	1	Tier 1
MONO C	SF3B6	0	0.1	0.3	0	0.5	0.9	Tier 2
Plasma	SNRPD2	0	0	0.3	0	0.5	0.8	Tier 2
CD4 SOX4	MARCHF7	0	0.3	0.5	0	0	0.8	Tier 2
CD4 ET	SLC28A2	0	0.1	0.1	0	0.5	0.7	Tier 2
MONO NC, NK R	VKORC1	0	0.1	0.1	0	0.5	0.7	Tier 2
MONO NC	UBE2Z	0	0.1	0	0.3	0	0.4	Tier 3
NK R	CMTR2	0	0	0.3	0	0	0.3	Tier 3
DC	SCML4	0	0.3	0	0	0	0.3	Tier 3
MONO NC	TIPARP‐AS1	0	0.3	0	0	0	0.3	Tier 3
CD4 ET	GABPB2	0	0.1	0	0	0	0.1	Tier 3
CD8 ET	ASTE1	0	0.1	0	0	0	0.1	Tier 3
CD8 NC	ZNF689	0	0.1	0	0	0	0.1	Tier 3

Abbreviations: DEG, differential expressed gene; MR, Mendelian randomization.

^a^ Repeating MR analysis using eQTLs with a stricter threshold of *p* < 5 × 10^−6^ as instrumental variables.

^b^ Significant colocalization was defined as PP.H4 > 0.8.

### 2.9 Ethics

All of the datasets used in our study can be publicly accessed without access to individual‐level information. These data are derived from studies on human subjects that have been approved by the relevant ethics committees.

## 3 Results

### 3.1 Single‐Cell Transcriptome‐Wide MR and Colocalization Analyses

**FIGURE 2:**
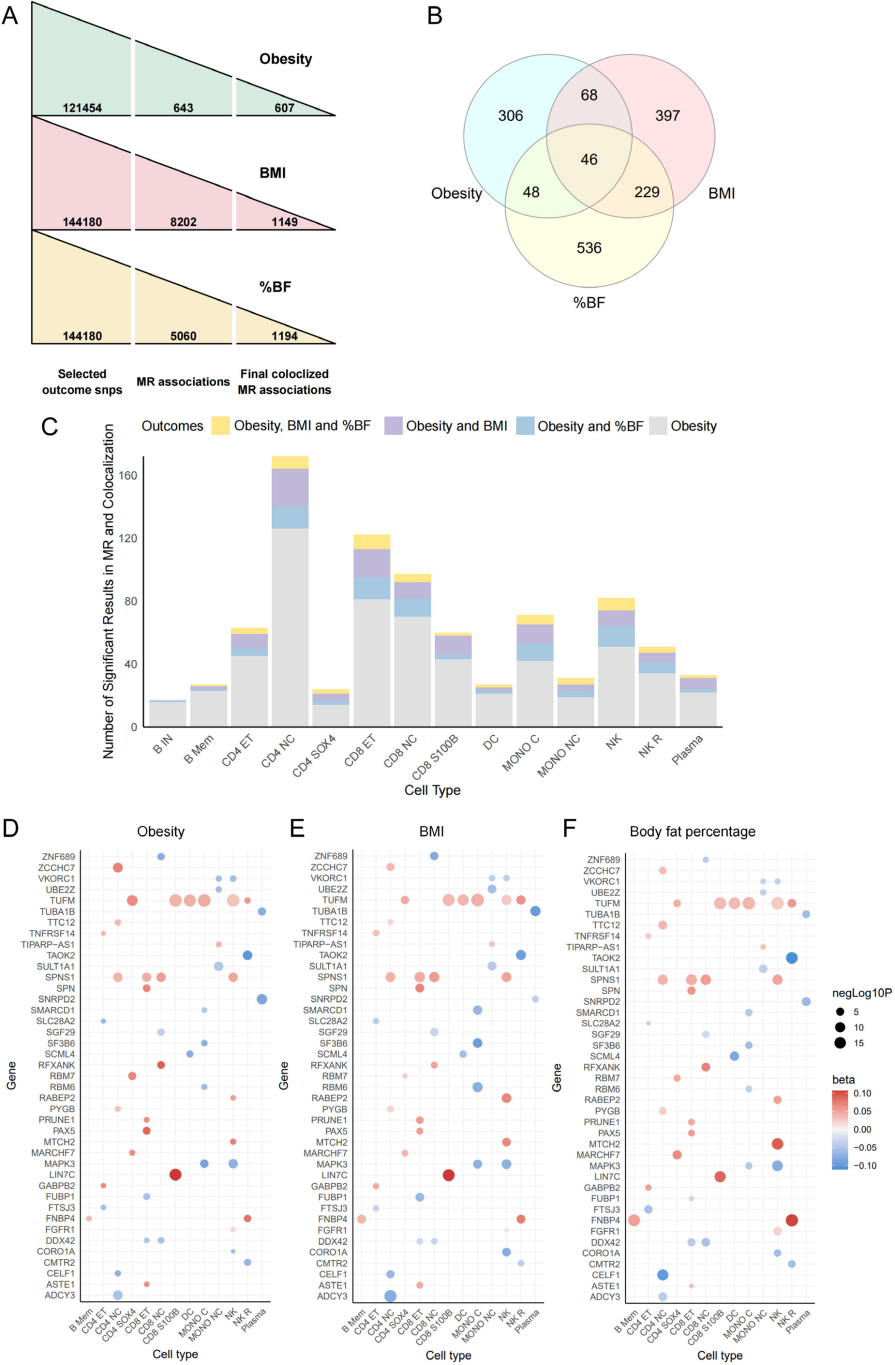
Summary of MR results of immune‐cell specific gene expressions on obesity‐related traits (A) Total number of selected SNPs, MR associations and colocalization for obesity, BMI and %BF. (B) Venn plot of the number of genes showing MR signals with obesity‐related traits. (C) Bar plot showing numbers of putative causal genes in 14 types of immune cells. (D–F) Bubble plot showing cell types, as well as beta and *p* values of MR results of the 41 replicable genes. %BF, body fat percentage; BMI, body mass index; MR, Mendelian randomization; SNP, single nucleotide polymorphism.

We used the eQTLs derived from single‐cell transcriptomes of 14 types of immune cells (B IN: immature B cells; B Mem: memory B cells; CD4 ET: CD4^+^ effector memory T cells; CD4 NC: CD4^+^ naive/central memory T cells; CD4 SOX4: CD4^+^ T cells expressing SOX4; CD8 ET: CD8^+^ effector memory T cells; CD8 NC: CD8^+^ naive/central memory T cells; CD8 S100B: CD8^+^ T cells overexpressing S100B; DC: dendritic cells; NK: natural killer cells; NK R: NK recruiting cells; Mono C: classical monocytes; Mono NC: nonclassical monocytes; Plasma: plasma cells) as instruments for the MR analysis. After LD clumping, *F*‐statistic testing and Steiger filtering, we selected 121,454, 144,180 and 144,180 eQTLs for MR analysis to estimate causal associations with obesity, BMI and %BF, respectively (Figure 2A). Using these eQTLs as IVs, we performed two‐sample MR and detected 643 gene‐obesity pairs, 8202 gene‐BMI pairs and 5060 gene‐%BF pairs showing robust evidence of causal relationships (FDR *p* < 0.05) (Figure 2A). Then we conducted a Cochran's *Q* test for MR associations estimated by two or more instruments to examine heterogeneity in the MR results, and removed the gene‐outcome pairs showing evidence of heterogeneity among instruments (*Q*‐value < 0.05). Genetic colocalization and LD check provided colocalization evidence (defined as (PP.H3 + PP.H4) > 0.8 and [PP.H4/(PP.H3 + PP.H4)] > 0.9 for colocalization, or *r* ^2^ ≥ 0.7 for LD check) of 607 MR signals for obesity, 1149 MR signals for BMI and 1194 MR signals for %BF (Figure 2A).

Our results indicated that 1239 genes were causally associated with only one obesity‐related outcome, whereas 391 genes had robust MR signals and colocalization evidence with two or more outcomes (Figure 2B). Forty‐six genes showed causal associations with all three outcomes and were considered replicable genes. The two‐sample MR results (including *p* value of FDR correction) of these 46 genes are summarized in Table S3. The genes with significant MR signals were enriched in CD4 NC, whereas CD4 NC, CD8 ET, Mono C and NK harboured relatively large numbers of replicable genes (Figure 2C). Among the 46 replicable genes, 41 had gene names corresponding to their ENSEMBL IDs, and their MR results are shown in Figure 2D–F. Most of these genes showed replicable MR signals in a single cell type, whereas *VKORC1*, *TUFM*, *SPNS1*, *MAPK3*, *FNBP4* and *DDX42* were found to be significantly associated with obesity‐related traits in two or more cell types (Figure 2D–F).

To assess the novelty of the 41 candidate genes, a literature search in PubMed and Web of Science databases was performed from their inception to November 2025, without restriction on regions, publication types, or languages. We employed a search strategy using the gene names and terms such as ‘obesity’, ‘body mass index’ and ‘body fat percentage’ to locate original articles, systematic reviews and meta‐analyses. We also searched for the genes in databases of gene‐disease associations including OMIM, Open Targets Platform, DrugBank and ChEMBL. Genes that had not been previously reported to be associated with obesity were defined as novel genes. In our study, 19 of the 41 candidate genes were defined as novel genes, whereas 22 had been found to be associated with obesity previously (defined as reported genes). The names of the 19 novel genes are shown in Table 1.

### 3.2 Pathway Enrichment of the Obesity‐Associated Genes

**FIGURE 3:**
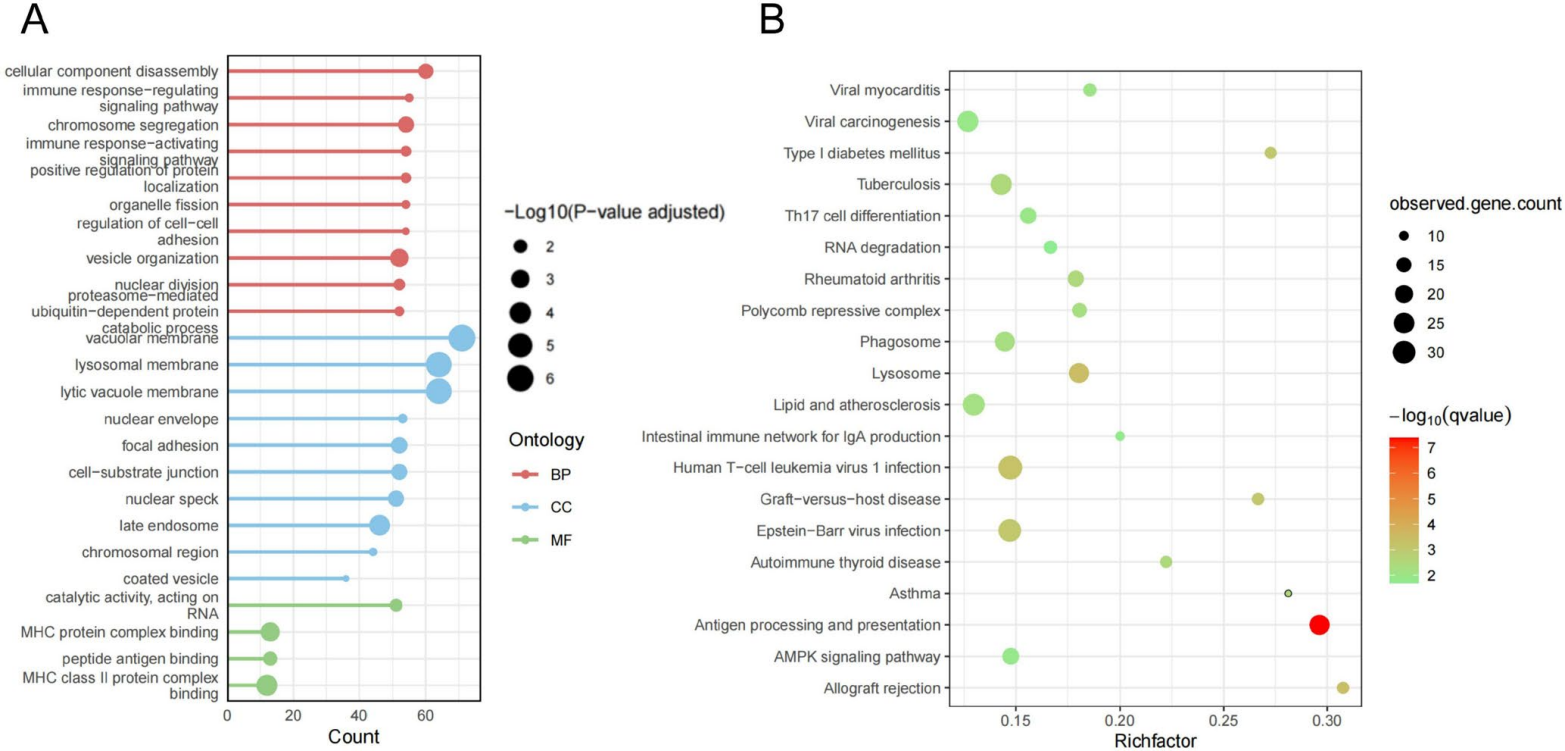
Functional enrichment results of the obesity‐related genes identified by MR and colocalization: (A) Gene Ontology (GO) enrichment results. (B) Kyoto Encyclopedia of Genes and Genomes (KEGG) enrichment results.

To explore the functional pathways enriched by the obesity‐associated genes, we performed functional enrichment analyses using GO and KEGG enrichment methods. Among the 1630 genes showing MR signals and colocalization evidence with one or more outcomes, 1494 genes could be mapped to ENTREZ IDs and were included in the enrichment analyses. The genes were enriched in several pathways including antigen processing and presentation, cellular component disassembly and catalytic activity (Figure 3).

### 3.3 scRNA‐Seq Analysis of PBMCs and Immune Cells Within SAT

**FIGURE 4:**
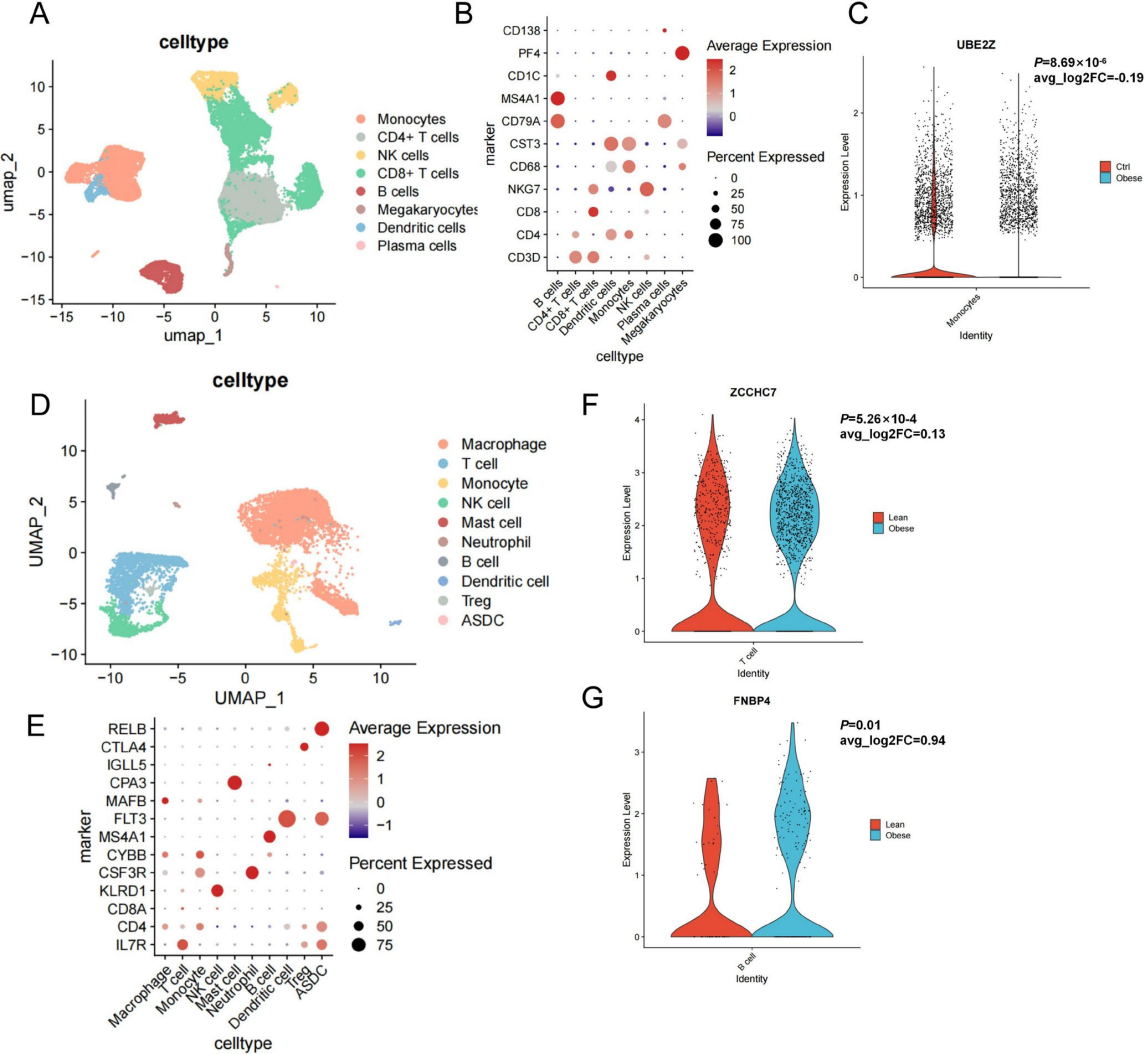
ScRNA‐seq and DEG analysis of putative causal genes (A, C) UMAPs of scRNA‐seq data of PBMCs (A) and immune cells within adipose tissue (C). (B, E) Dotplots showing expressions of marker genes of PBMCs (B) and immune cells within adipose tissue (E). (C, F, G) Violin plots of genes with differential expression levels consistent with MR results of monocytes (C), T cells (F) and B cells (G). DEG, differential expressed genes. PBMC, peripheral blood mononuclear cell; scRNA‐seq, single‐cell RNA sequencing; UMAP, uniform manifold approximation and projection.

We performed scRNA‐seq analysis to assess the expressions of our replicable genes in another scRNA‐seq dataset of PBMCs and in SAT from lean and obese volunteers. For PBMCs, a total of 41,280 cells from 20 samples were analyzed. We referred to the marker genes of immune cells described previously for annotation [26, 27], and classified the cells into eight types: B cells, CD4^+^ T cells, CD8^+^ T cells, dendritic cells, monocytes, NK cells, plasma cells and megakaryocytes (Figure 4A,B). The DEG analysis revealed a significantly decreased expression level of *UBE2Z* in monocytes of the obese group compared with the lean group (*p* = 8.69 × 10^−6^), which is directionally consistent with the MR signals (Figure 4C). For the immune cells within SAT, 10,862 cells from eight SAT samples were included in our analysis and were classified into 10 types according to the original literature [25] including macrophages, T cells, monocytes, NK cells, mast cells, neutrophils, B cells, dendritic cells, Tregs and adipose‐derived stem cells (ASDCs) (Figure 4D,E). The expression levels of *ZCCHC7* in T cells (*p* = 5.26 × 10^−4^) and *FNBP4* in B cells (*p* = 0.01) showed significant changes in line with their MR results (Figure 4F,G).

### 3.4 Repurposing of Drug Targets

To clarify the clinical potential of the obesity‐related genes, we searched for drugs that target the 41 replicable genes on OpenTargets, DrugBank, Drug Gene Interaction database, Pharos and the literature of related drug trial studies. Six drugs targeting these candidate genes were identified, as summarized in Table 2. For all six drugs, the directions of pharmacologic effects on the corresponding genes align with the direction of MR effect. Two of the six drugs act on the novel genes that had not been reported in previous studies on obesity (*PYGB*, *PRUNE1*). Five out of the six putative causal genes only showed MR associations in one specific cell type, whereas one gene (*TUFM*) showed MR associations in more than two cell types. Among the 19 novel genes, besides the two target genes with existing drugs, six genes (*FTSJ3*, *DDX42*, *SF3B6*, *SNRPD2*, *SLC28A2* and *VKORC1*) showed evidence of druggability in the databases mentioned above, whereas the other 10 genes lacked evidence of druggability.

**TABLE 2:** Potential drug target genes and their related drugs with repurposing evidence.

Gene type	Gene	Cell type	Drug name	Molecular action	Current clinical indication
Novel	PYGB	CD4 NC	PSN357	Inhibitor	Diabetes
	PRUNE1	CD8 ET	Dipyridamole	Inhibitor	Platelet aggregation inhibition
Previous reported	TUFM	CD4 SOX4, CD8 S100B, DC, MONO C, NK, NK R	Gilteritinib	Inhibitor	Acute myeloid leukaemia
	FGFR1	NK	Infigratinib	Inhibitor	Cholangiocarcinoma
	TUBA1B	Plasma	Davunetide	Modulator	Cognitive impairment

### 3.5 Tier System and Prioritization of Drug Targets for Obesity

**FIGURE 5:**
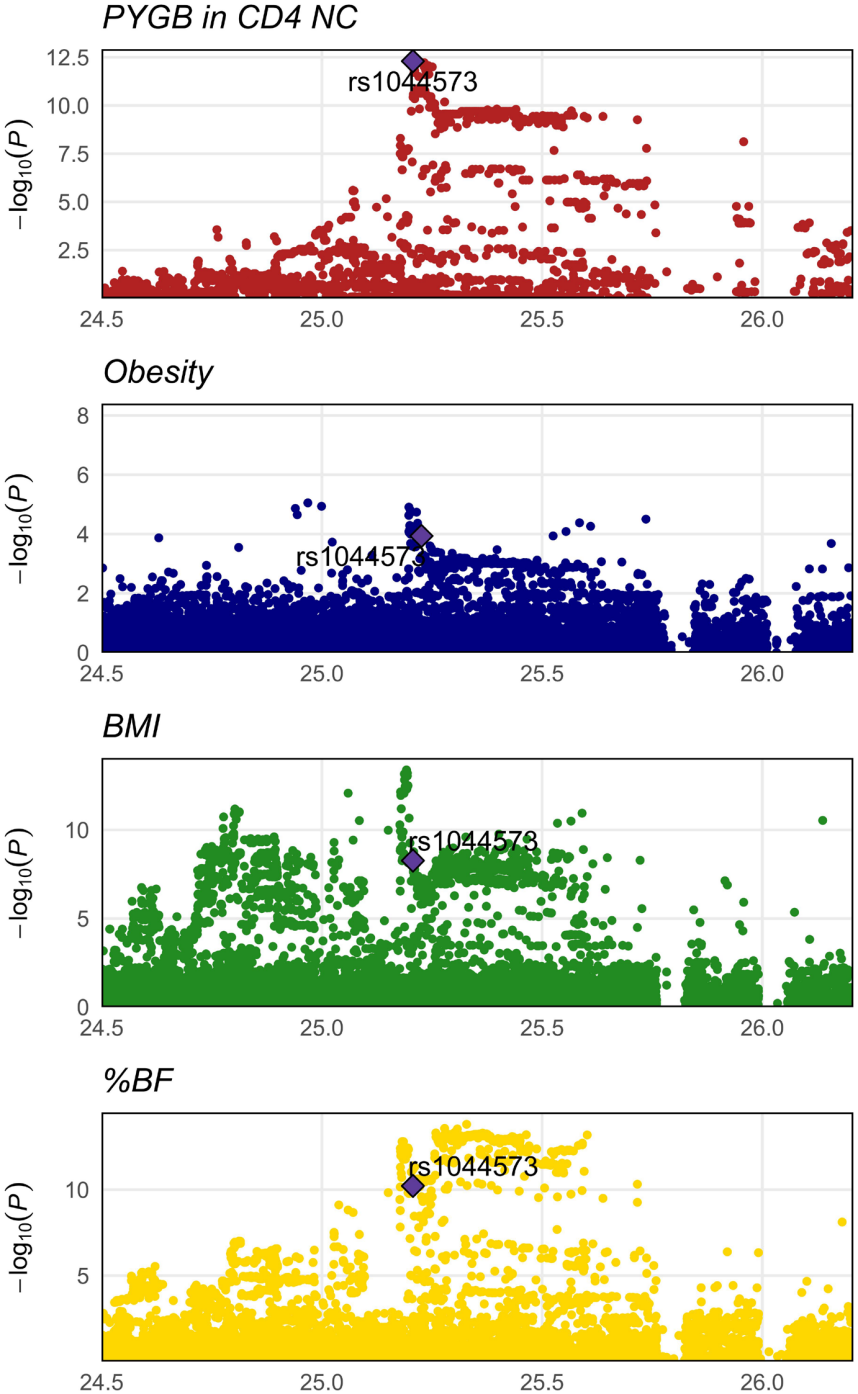
LocusZoom plots illustrating evidence of genetic colocalization between the cell‐specific expressions of the top gene identified by Tier system and the three obesity‐related outcomes in the locus of *PYGB* in CD4^+^ naive/central memory T cells.

We developed a Tier system combining sensitivity analyses, pathway enrichment, DEG analysis, and druggable evidence to evaluate the potential of the obesity‐associated genes as drug targets. Results of sensitivity analyses of MR and colocalization analyses involved in this Tier system are listed in Tables S4 and S5. The 19 novel genes were involved in the scoring and ranked based on their total scores in the Tier system. Our results demonstrated that seven genes were ranked as Tier 1 drug targets (overall score ≥ 1), five genes were ranked as Tier 2 targets (overall score between 0.5 and 1) and the remaining seven targets were ranked as Tier 3 targets (Table 1). *PYGB* (glycogen phosphorylase, brain form) in CD4 NC had the highest score of 2.3, suggesting relatively strong evidence of drug target for obesity. LocusZoom plots illustrating the associations of variants at the *PYGB* loci with obesity, BMI and %BF are shown in Figure 5. The sensitivity analysis on the Tier system is summarized in Table S6. The majority of top‐ranked genes were consistently retained across alternative models, with Spearman's *ρ* of 0.968 (Rank_original vs. Rank_equal) and 0.978 (Rank_original vs. Rank_downweight), showing the stability of the scoring system.

## 4 Discussion

Previous GWAS and MR studies have identified associations of tissue‐level gene expression and obesity using bulk eQTL data [29, 30]. Only a small number of studies focused on particular cell types in which gene expression may play a role in the functional regulation. Therefore, the effects of cell‐specific gene expression on the outcomes are often obscured. Hao et al. [31] used sc‐eQTL from different brain cells to identify the associations between cell‐specific gene expression and obesity. Besides the central nervous system, the immune and metabolic processes in blood and peripheral organs are also potential targets for obesity treatment. Herein, we utilized the sc‐eQTL data from the OneK1K database, and estimated the causal relationship between cell‐specific gene expression in 14 immune cell types and the obesity‐related traits. We have tested two‐sample MR and colocalization on obesity‐related traits from three different GWAS summary datasets and detected 1630 genes associated with at least one outcome, of which 46 genes were associated with all three outcomes. The results of MR and colocalization analyses highlight the enrichment of eQTLs in some specific cell types such as CD4 NC, CD8 ET, Mono C and NK. For pathway enrichment, we found that antigen processing and presentation, cellular component disassembly, and catalytic activity were enriched by the identified genes. Using scRNA‐seq data of the PBMCs and the immune cells within SAT, we compared the celltype‐specific expression levels of the putative causal genes between lean and obese individuals, and observed consistent results with MR in the expressions of *UBE2Z* in monocytes, *ZCCHC7* in T cells, and *FNBP4* in B cells. To integrate our results from MR and colocalization analyses, functional enrichment, scRNA‐seq analysis and drug repurposing, we applied a Tier system to the prioritization of potential drug targets for obesity, and identified *PYGB* as the top drug target gene with the highest score.

There is mounting evidence on the critical role of immune cells in the pathogenesis and development of obesity. A study using spatially resolved single‐nucleus analysis revealed higher proportions of CD4^+^ and CD8^+^ T cells, NK cells and B cells in obese AT, suggesting the aggravated lymphocyte infiltration [32]. In our study, the enrichment of obesity‐related genes in CD4 NC indicates that circulating CD NC may potentially be involved in the mechanisms of obesity. Previous studies have demonstrated that CD4^+^ T cells control immune‐AT crosstalk in the pathogenesis of obesity: pro‐inflammatory CD4^+^ T cells impair adipocyte function by secreting cytokines and chemokines, whereas anti‐inflammatory CD4^+^T cells promote the function of adipocytes [33]. Zou et al. [34] reported an accelerated weight regain in male recipient mice that underwent adoptive transfer of T helper (Th) cells from obese male mice, indicating that obesity‐related memory can be imprinted in immune cells and stored within CD4^+^ T cells. Although adipose tissue‐resident CD4^+^ T cells have been proposed to be a therapeutic target for obesity and obesity‐related disorders, little is known about whether CD4^+^ T cells in peripheral blood are involved in the incidence and development of obesity. In humans, homeostatic proliferation of circulating CD4^+^ T cells is increased in individuals with obesity [35], but the underlying mechanism and the causal link between changes in circulating CD4^+^ T cells and obesity remain unclear. Therefore, our findings may contribute to the understanding of the mechanisms in which immune cells are involved in obesity pathogenesis, as well as the potential of immune cells in weight‐loss treatment. In addition, monocytes have also been observed to be involved in obesity and weight regain. White adipose tissue (WAT) is infiltrated by large numbers of peripheral blood monocytes in obese individuals, and some of the latest studies on obesity therapy focus on the monocyte connection in immune responses, highlighting the alterations in the adipocyte component of the bone marrow in the context of obesity that induces monocytosis and migration of monocytes to adipose tissue [36]. Zhou et al. [37] reported the suppressed weight regain in mice models that underwent adoptive transfer of CD7^+^ monocytes, which is attributed to the preferential migration to adipose tissue and the induction of WAT beiging through the FGL2/PKA pathway.

In our study, three obesity‐related traits were selected as outcomes for MR analysis including obesity, BMI and %BF. BMI, the most frequently used diagnostic criterion for obesity, is calculated as weight in kilograms divided by height in metres squared (kg/m^2^) [38]. BMI is easy to obtain, but it does not distinguish fat mass from lean mass. Body fat percentage, by contrast, reflects body composition to help detect the excessive adipose accumulation [39]. Therefore, genes showing MR signals and colocalization evidence with all three obesity‐related traits were regarded as replicable genes for further analysis.

Twenty‐two of the 41 replicable genes with identifiable gene names have been reported to be associated with obesity in previous studies. Notably, we have detected replicable MR signals of ADCY3, a gene that has been extensively accepted as a therapeutic target for obesity [40], supporting the reliability of our MR results. We also identified previously unrecognized obesity risk associations of 19 genes. Most of these genes showed causal relationships with obesity exclusively in one cell type. For example, our results indicated that leukosialin (*SPN*, also called *CD43*) expression in CD8 ET, rather than other PBMCs, drives the risk of the three obesity‐related traits. *SPN* is a major glycoprotein of T lymphocytes that participates in T‐cell‐B‐cell interaction and binds to ligands on antigen‐presenting B cells, and it is also involved in the activation of T cell adhesion [41, 42].

In our Tier system, PYGB in CD4 NC was assigned the highest score of 2.3, indicating its potential as a drug target for obesity treatment. *PYGB* has been validated to promote glycogenolysis and release of glucose‐6‐phosphate in memory T cells, contributing to their activation during the early recall response [43]. ZCCHC7 in CD4 NC, DDX42 in CD8 ET and CD8 NC, and PRUNE1 in CD8 ET scored 1.6, 1.6 and 1.4, respectively. *DDX42* participates in intrinsic and innate immunity and was observed to be inversely associated with infiltration scores of various activated immune cells within carotid plaques [44]. A pharmacogenomics study found that *ZCCHC7* may be involved in the interferon signalling in response to interferon‐beta in patients with relapsing–remitting multiple sclerosis [44]. *PRUNE1*, a gene that drives polarization of tumour‐associated macrophages, is associated with cancer metastasis [45, 46]. These previous studies indicate that the top genes in our Tier system play potential roles in immune‐related pathways of diseases. The underlying mechanisms of these obesity‐related processes and the pathogenesis of obesity still need to be further elucidated. Nevertheless, the pathway enrichment of the genes with MR signals indicates an involvement of antigen processing and presentation in obesity, which may be worth exploring in future research. It is also worth noting that the causal genes detected in our study are also enriched in pathways of some common complications and comorbidities of obesity such as atherosclerosis, autoimmune thyroid disease and asthma. Therefore, the mechanisms of obesity involving the identified genes may also contribute to these obesity‐related diseases.

In recent years, some immunomodulatory mechanisms and therapies have demonstrated the anti‐obesity efficacy. Leptin receptor‐positive sympathetic perineurial barrier cells preserve body weight homeostasis by mediating the inflammation of the brown AT environment and promoting thermogenesis [47]. GMI, an immunomodulatory protein cloned from *Ganoderma microsporum*, has been reported to ameliorate sarcopenic obesity through activating DNAJA3 to enhance mitochondrial homeostasis [48]. Herein, we searched for drugs targeting the 41 replicable genes in our study. PSN357, the drug targeting PYGB, is a glycogen phosphorylase inhibitor designed for the treatment of T2D [49]. A study on multiple myeloma demonstrated that PRUNE1 may be inhibited by dipyridamole [50], which is an anti‐platelet agent commonly used in cardiovascular diseases (CVD). Considering that obesity is commonly comorbid with T2D and CVD, research on the application of these drugs in obesity treatment is warranted.

Our study has some limitations. First, our MR analysis used sc‐eQTL data only from immune cells in circulating blood, not from those in AT, which limited our ability to investigate the potential causal associations between gene expression in adipose‐tissue–resident immune cells and the pathogenesis of obesity. It is necessary to develop accessible datasets of cell‐type‐specific eQTLs in AT, which will facilitate studies on cell‐specific drug targets in AT. The OneK1K PBMC sc‐eQTL dataset represents immune cells profiled under steady‐state, ex vivo conditions without external stimulation. Although the immature/naive memory cells and memory cells were defined by specific markers, studies on eQTLs of dynamic activation states are needed to improve the precision of drug target identification based on the different gene expression profiles in different activation states of immune cells. Furthermore, sc‐eQTL data reveal gene expression rather than protein expression. Considering that gene expression typically functions at the protein level and that the level of gene expression is not always consistent with encoded proteins, cell‐specific pQTL MR will further facilitate studies on functional validation and druggability translation of the cell‐specific eQTLs. In addition, our colocalization analysis, assuming a single causal variant within each locus, may not hold in regions with complex LD structure or multiple independent signals, which may lead to biased evidence of colocalization. Future studies utilizing conditional or fine‐mapping‐based colocalization approaches could better resolve multiple causal signals and improve inference. Another problem is the limitation of generalizability that arose from the fact that most participants from the OneK1K dataset were of Northern European ancestry, and multi‐ancestry replication is necessary for the translation of target genes. Further studies are warranted to ascertain the mechanisms underlying the associations between immune cells and obesity, as well as the efficacy and safety of the potential drug targets detected by our study. For instance, cell‐type–specific perturbation experiments, as well as pharmacologic testing via in vitro and in vivo experiments, will be essential in future work to confirm the biological mechanisms and therapeutic effects of the prioritized targets identified in this study.

## 5 Conclusions

In conclusion, we assessed causal relationships between gene expressions in 14 immune cell types and the obesity‐related traits through MR and colocalization analyses, and evaluated their potential as drug targets for obesity treatment through a Tier system. We hope that these findings will provide evidence for the role of immune cells in the pathogenesis and development of obesity, and facilitate therapeutic target selection for obesity.

## Author Contributions

**Yong Tian:** data curation, formal analysis, investigation, writing – original draft. **Xiaoying Ren:** formal analysis, investigation, writing – original draft, visualization. **Juan Tian:** investigation, formal analysis, visualization, writing – original draft. **Guang Wang:** conceptualization, funding acquisition, writing – review and editing, supervision, project administration. **Xingjian Zhang:** data curation, investigation, formal analysis, writing – original draft, visualization. **Jia Liu:** conceptualization, funding acquisition, writing – review and editing, supervision, project administration.

## Funding

This work was supported by a grant from the Beijing Natural Science Foundation (No. L248018), the National Key R&D Program of China (No. 2022YFA0806400), the National Natural Science Foundation of China (No. 82470889) and the Beijing Hospitals Authority's Ascent Plan (No. DFL20220302).

## Ethics Statement

The authors have nothing to report.

## Consent

The authors have nothing to report.

## Conflicts of Interest

The authors declare no conflicts of interest.

## Supporting information


**Table S1:** Clinical and demographic information of donors in the scRNA‐seq data of PBMCs analysed in our study.
**Table S2:** BMI of donors in the scRNA‐seq data of immune cells in the subcutaneous white adipose tissue analysed in our study.
**Table S3:** MR results of 46 genes causally associated with all 3 obesity‐related outcomes (obesity, BMI and %BF).
**Table S4:** MR results for candidate genes with a stricter threshold of the eQTLs selected as instrumental variables (*p* < 5e‐06).
**Table S5:** Colocalization results for candidate genes with robust coloc evidence (PP.H4 > 0.8).
**Table S6:** Sensitivity analyses of the Tier system with alternative scoring schemes (equal weights and down‐weight annotation).

## Data Availability

The single‐cell eQTL data of 14 immune cells was downloaded from the OneK1K database (https://onek1k.org). Data used for scRNA‐seq analysis was downloaded from the CZ CELLxGENE (https://cellxgene.cziscience.com) and the Gene Expression Omnibus (GEO) database (GSE176067).
